# Association between MASLD and increased risk of serious bacterial infections requiring hospital admission: A meta‐analysis

**DOI:** 10.1111/liv.16101

**Published:** 2024-09-11

**Authors:** Alessandro Mantovani, Riccardo Morandin, Veronica Fiorio, Maria Giovanna Lando, Alberto Gaviraghi, Leonardo Motta, Federico Gobbi, Herbert Tilg, Christopher D. Byrne, Giovanni Targher

**Affiliations:** ^1^ Department of Medicine, Section of Endocrinology, Diabetes and Metabolism University of Verona Verona Italy; ^2^ Department of Infectious Tropical Diseases and Microbiology IRCCS Sacro Cuore‐Don Calabria Hospital Negrar di Valpolicella Italy; ^3^ Department of Clinical and Experimental Sciences University of Brescia Brescia Italy; ^4^ Department of Internal Medicine I, Gastroenterology, Hepatology, Endocrinology and Metabolism Medical University Innsbruck Innsbruck Austria; ^5^ National Institute for Health and Care Research, Southampton Biomedical Research Centre University Hospital Southampton and University of Southampton Southampton UK; ^6^ Department of Medicine University of Verona Verona Italy; ^7^ Metabolic Diseases Research Unit IRCCS Sacro Cuore‐Don Calabria Hospital Negrar di Valpolicella Italy

**Keywords:** MASLD, meta‐analysis, metabolic dysfunction‐associated steatotic liver disease, NAFLD, non‐alcoholic fatty liver disease, serious bacterial infections

## Abstract

**Background:**

Previous studies have reported an association between metabolic dysfunction‐associated steatotic liver disease (MASLD) and the risk of serious bacterial infections. However, the magnitude of the risk and whether this risk varies with the severity of MASLD remains uncertain. We performed a meta‐analysis of observational studies to quantify the association between MASLD and serious bacterial infections requiring hospital admission.

**Methods:**

We systematically searched PubMed, Scopus, Web of Science and Embase from database inception to 1 April 2024, using predefined keywords to identify studies examining the risk of serious bacterial infections among individuals with and without MASLD. MASLD was diagnosed using liver biopsy, imaging or International Classification of Diseases codes. Meta‐analysis was performed using random‐effects modelling.

**Results:**

We identified six cross‐sectional and two prospective cohort studies with aggregate data on ~26.6 million individuals. MASLD was significantly associated with higher odds of serious bacterial infections (pooled random‐effects odds ratio 1.93, 95% confidence interval [CI] 1.44–2.58; *I* ^*2*^ = 93%). Meta‐analysis of prospective cohort studies showed that MAFLD was associated with an increased risk of developing serious bacterial infections (pooled random‐effects hazard ratio 1.80, 95% CI 1.62–2.0; *I* ^*2*^ = 89%). This risk further increased across the severity of MASLD, especially the severity of fibrosis (pooled random‐effects hazard ratio 2.42, 95% CI 1.89–2.29; *I* ^*2*^ = 92%). These results remained significant after adjusting for age, sex, obesity, diabetes and other potential confounders. Sensitivity analyses did not modify these findings. The funnel plot did not reveal any significant publication bias.

**Conclusions:**

This meta‐analysis shows a significant association between MASLD and an increased risk of serious bacterial infections requiring hospital admission.

**Abbreviations**
HR: hazard ratio
MASH: metabolic dysfunction‐associated steatohepatitis
MASLD: metabolic dysfunction‐associated steatotic liver disease
MDR: multidrug resistant
NAFLD: non‐alcoholic fatty liver disease
NOS: Newcastle‐Ottawa scale
OR: odds ratio

**Key points**

This comprehensive meta‐analysis of observational studies shows for the first time that metabolic dysfunction‐associated steatotic liver disease (MASLD) is significantly associated with an increased risk of having or developing serious bacterial infections requiring hospital admission. Further well‐designed prospective and mechanistic studies are needed to better understand the link between MASLD and the risk of developing serious bacterial infections.

## 1 INTRODUCTION

Non‐alcoholic fatty liver disease (NAFLD), recently renamed metabolic dysfunction‐associated steatotic liver disease (MASLD), has become the leading cause of chronic liver disease globally. The recently proposed change in nomenclature from NAFLD to MASLD responds to our better understanding of the pathophysiological processes that lead to the development and progression of MASLD (where metabolic dysfunction plays a key role in the pathophysiology of this common liver disease).^1^ The global prevalence of MASLD is estimated to be around 38% among adult individuals and is projected to continue to rise with increasing global rates of obesity and type 2 diabetes.^1^, ^2^, ^3^

MASLD poses a significant health and economic burden worldwide.^4^, ^5^ Substantial epidemiological evidence indicates that MASLD is a multisystem disease^6^, ^7^ that is associated not only with liver‐related complications (cirrhosis, end‐stage liver disease and hepatocellular carcinoma) but also with an increased risk of developing extrahepatic cancers (mainly non‐liver gastrointestinal cancers)^8^ and cardiometabolic diseases, such as new‐onset type 2 diabetes,^9^ fatal and non‐fatal cardiovascular events^10^ and adverse renal outcomes.^11^

Among the least investigated MASLD‐related extrahepatic complications, an ever‐increasing number of observational cross‐sectional and prospective studies have assessed the association between MASLD and the risk of severe bacterial infections, such as pneumonia, meningitis, sepsis, gastrointestinal/abdominal infections or other bacterial infections requiring hospital admission (as extensively discussed below). However, the magnitude of this risk and whether the risk varies with the severity of MASLD remain uncertain.

Therefore, we have performed a comprehensive systematic review and meta‐analysis of relevant observational studies to quantify the magnitude of the association between MASLD and the risk of serious bacterial infections requiring hospital admission. Moreover, we have also examined whether there was an association between the severity of MASLD and the risk of serious bacterial infections. Clarifying the magnitude of the risk of serious bacterial infections requiring hospital admission in patients with MASLD might favour the development of prevention strategies for serious bacterial infections in this patient population.

## 2 MATERIALS AND METHODS

### 2.1 Registration of review protocol

The protocol of this systematic review was registered on Open Science Framework (registration DOI: https://doi.org/10.17605/OSF.IO/B7TDG).

### 2.2 Data sources and searches

This systematic review has been performed following the Preferred Reporting Items for Systematic Reviews and Meta‐Analyses (PRISMA) and the Meta‐analysis Of Observational Studies in Epidemiology (MOSE) guidelines.^11^, ^12^ We systematically searched PubMed, Web of Science, Embase and Scopus from database inception to 1 April 2024, to identify relevant observational studies examining the association between MASLD and the risk of serious bacterial infections requiring hospital admission. Search free text terms were ‘serious bacterial infections’ OR ‘severe bacterial infections’ OR ‘serious/severe infections’ OR ‘bacterial infections’ OR ‘infectious diseases’ AND ‘fatty liver’ OR ‘nonalcoholic fatty liver disease’ OR ‘NAFLD’ OR ‘nonalcoholic steatohepatitis’ OR ‘NASH’ OR ‘metabolic dysfunction‐associated steatotic liver disease’ OR ‘MASLD’ OR ‘metabolic dysfunction‐associated steatohepatitis’ OR ‘MASH’. Searches were restricted to human studies and English language studies. Subjects included in the meta‐analysis were of either sex without age, race or ethnicity restrictions.

### 2.3 Study selection

The inclusion criteria of the meta‐analysis were as follows: (1) observational (cross‐sectional, case–control or longitudinal) studies examining the risk of serious bacterial infections requiring hospital admission among adult (age ≥18 years) individuals with and without MASLD (or NAFLD); (2) studies that reported odds ratios (ORs) or hazard ratios (HRs) with 95% confidence intervals (95% CIs) values for the outcome of interest; (3) the diagnosis of MASLD (or NAFLD) was based on liver biopsy, imaging techniques, blood‐based biomarkers/scores or International Classification of Diseases (ICD) codes, in the absence of significant alcohol consumption (usually defined as <20 g/day for women and <30 g/day for men) or other competing causes of hepatic steatosis (e.g. viral hepatitis, iron overload and use of potentially hepatotoxic drugs); and (4) the diagnosis of serious bacterial infections, such as pneumonia, meningitis, sepsis, gastrointestinal/abdominal infections or other severe bacterial infections requiring hospital admission was based on hospital charts, electronic medical records or ICD‐9/ICD‐10 codes (as specified in each eligible study).

The exclusion criteria of the meta‐analysis were as follows: (1) congress abstracts, case reports, reviews, practice guidelines or commentaries; (2) studies in which the identification of MASLD (or NAFLD) was based exclusively on serum aminotransferase concentrations; (3) studies which did not exclude individuals with significant alcohol consumption or other known causes of chronic liver disease; (4) studies that examined the association between MASLD (or NAFLD) and the risk of non‐severe bacterial infections not requiring hospital admission (e.g. prior or active *Helicobacter pylori* infection or periodontitis); and (5) studies examining the association between MASLD (or NAFLD) and the risk of more severe COVID‐19 infection or other types of infections (viral, fungal or parasitic).

### 2.4 Data extraction and quality assessment

Data from studies eligible for the aggregate data meta‐analysis were independently extracted by two investigators (AM and GT). Any disagreements between investigators about including eligible studies were resolved by consensus and a third investigator if needed (RM).

For each eligible study, we extracted data on publication year, study design, sample size, country, population characteristics, methodologies used for the diagnosis of MASLD and serious bacterial infections, severity of MASLD, outcomes of interest, matching and confounding factors included in multivariable regression analyses and length of follow‐up (for longitudinal studies). In the case of multiple publications of the same database, we included the most up‐to‐date or comprehensive information.

The overall quality of the studies included in the aggregate data meta‐analysis was assessed using the Newcastle‐Ottawa scale (NOS) by two independent authors (AM and GT). Any disparities in scoring were reviewed, and consensus was obtained following discussion. The NOS scale is a validated scale for non‐randomized studies in meta‐analyses, which uses a star system to assess the quality of a study in three domains: selection, comparability and outcome/exposure. The NOS assigns a maximum of four stars for selection (or five stars in the case of cross‐sectional studies), two for comparability, and three for outcome/exposure. We judged studies that received a score of at least eight stars to be at low risk of bias, thus reflecting the highest quality.

### 2.5 Data synthesis and analysis

The primary outcome measure of the meta‐analysis was the presence of serious bacterial infections, such as pneumonia, meningitis, sepsis, urinary tract infections, gastrointestinal/abdominal infections or other bacterial infections requiring hospital admission for cross‐sectional studies or the risk of developing incident serious bacterial infections requiring hospital admission over the follow‐up for longitudinal studies. The ORs (for cross‐sectional studies) or HRs (for longitudinal studies) and their 95% CIs were considered as the effect size for all the eligible studies. When studies reported ORs/HRs with varying degrees of covariate adjustment, we extracted those that reflected the maximum extent of adjustment for potentially confounding factors. The adjusted ORs/HRs of all eligible studies were pooled, and an overall effect‐size estimate was calculated using a random‐effects model since high heterogeneity was expected for a meta‐analysis of observational studies.

The statistical heterogeneity among studies was evaluated by the chi‐squared test and the *I* ^*2*^‐statistic, which estimates the percentage of variability across studies due to heterogeneity rather than chance alone. The proportion of heterogeneity accounted for by between‐study variability was assessed using the *I* ^*2*^‐statistic and adjudicated to be significant if the *I* ^*2*^ index was >50%.^13^ The possibility of publication bias was examined using the visual inspection of funnel plots and the Egger's regression asymmetry test.^14^

To explore the possible sources of (expected) high heterogeneity among the included studies and test the robustness of the observed associations, we performed subgroup analyses by study country, study design and diagnostic methods used for identifying MASLD. We also tested for possible excessive influence of individual studies using a meta‐analysis influence test that eliminated each included study at a time. Finally, we performed univariable meta‐regression analyses to test the impact of age, sex, body mass index (BMI) and percentage of established diabetes on the effect size for the association between MASLD and the risk of serious bacterial infections.

All statistical tests were two‐sided and a *p‐*value <.05 was considered significant. We used R version 4.3.3 (R Core Team 2023, R Foundation for Statistical Computing, Vienna, Austria. https://www.R‐project.org/) for all statistical analyses with the following packages: *meta* (version 7.0–0) and *metafor* (version 4.4‐0).

## 3 RESULTS

### 3.1 Study selection and characteristics

The PRISMA flow diagram summarizes the search and selection processes of the meta‐analysis (Figure S1). After examining the titles and abstracts of the publications and excluding duplicates, we identified 12 potentially eligible studies from four large electronic databases (PubMed, Scopus, Embase and Web of Science) from the inception to 1 April 2024. We excluded four observational studies because of unsatisfactory outcome measures (as specified in Table S1). Consequently, we identified eight unique observational studies (six cross‐sectional and two prospective cohort studies) for the final inclusion in the meta‐analysis.

The main characteristics of these selected observational studies are shown in Table 1.^15^, ^16^, ^17^, ^18^, ^19^, ^20^, ^21^, ^22^ Overall, the six cross‐sectional hospital‐based studies included 26 434 377 individuals hospitalized for serious bacterial infections (43.6% men; mean age 60 years; ~36% had a diagnosis of MASLD). The diagnosis of MASLD was based on liver ultrasonography and/or computed tomography (*n* = 5 studies) and ICD‐10 codes (*n* = 1 study). No cross‐sectional studies were available on using liver biopsy to diagnose MASLD. Four studies were conducted in Israel, one in the USA and one in Croatia. Four of these cross‐sectional six studies obtained seven stars and two studies obtained six stars on the NOS scale, thus reflecting a moderately high risk of bias. As shown in Table 1, the two eligible prospective cohort studies were carried out in Sweden and included 221 663 middle‐aged individuals (52% men; mean age 55 years) followed for a median period of 14.1 and 5.3 years, respectively. The diagnosis of MASLD was based on liver biopsy in one cohort study and ICD codes in the other. The two cohort studies obtained at least seven stars on the NOS scale, thus reflecting a relatively low risk of bias.

**TABLE 1:** Eligible cross‐sectional (*n* = 6) and prospective studies (*n* = 2) examining the association between MASLD and the risk of having or developing serious bacterial infections requiring hospital admission.

Authors, Year (ref.)	Study design	Study participants	MASLD diagnosis, % of cases^a^	Outcome measure, % of cases	Covariate adjustments	Study results	NOS
*Cross‐sectional studies*							
Nseir et al., 2011^15^	Cross‐sectional hospital‐based study (retrospective analysis)	347 Israeli hospitalized adult individuals (M/F: 159/188, mean age: 52 years, mean BMI: 29.5 kg/m^2^, % diabetes: 38%)	Ultrasound, 70% (*n* = 247)	Recurrent bacterial infections over 3 years (urinary tract infections, upper or lower respiratory tract infections or soft tissue infections), 17.6% (*n* = 61)	Age, obesity, diabetes, metabolic syndrome, waist circumference, HOMA‐IR, serum C‐reactive protein, serum MDA, serum ALT, serum PON‐1 activity, and serum 25(OH)D levels	MASLD was associated with higher risk of recurrent bacterial infections, especially urinary tract infections (aOR 3.0, 95% CI 2.6–4.2)	7
Nseir et al., 2017^16^	Hospital‐based case–control study (retrospective analysis)	141 Israeli hospitalized adult individuals with community‐acquired pneumonia and 141 hospitalized individuals without community‐acquired pneumonia matched for age, sex and BMI (M/F: 180/102, mean age: 63 years, mean BMI: 30 kg/m^2^, % diabetes: 50%)	Ultrasound or computed tomography, 34% (*n* = 96)	Community‐acquired pneumonia, 50% (*n* = 141)	Age, sex, BMI, smoking history, diabetes, congestive heart failure and chronic obstructive pulmonary disease	MASLD was associated with higher risk of community‐acquired pneumonia (aOR 2.50, 95% CI 2.0–3.15)	7
Nseir et al., 2019^17^	Hospital‐based case–control study (retrospective analysis)	186 Israeli hospitalized premenopausal women with acute urinary tract infections and sepsis and 186 premenopausal women without acute urinary tract infections or sepsis matched for age, sex and BMI (M/F: 0/372, mean age: 40 years, mean BMI: 30.5 kg/m^2^, % diabetes: NA)	Ultrasound, 32.5% (*n* = 121)	Recurrent urinary tract infections over 3 years, 50% (*n* = 186)	Age, obesity, maternal history of recurrent urinary tract infections, no‐use of probiotics, sexual intercourse and serum 25(OH)D levels	MASLD was associated with higher risk of recurrent urinary tract infections (aOR 1.60, 95% CI 1.3–2.0) in premenopausal women	7
Nseir et al., 2020^18^	Hospital‐based case–control study (retrospective analysis)	115 Israeli hospitalized adult individuals with Clostridium difficile‐associated diarrhoea and 115 hospitalized individuals without Clostridium difficile‐associated diarrhoea matched for age, sex and BMI (M/F: 134/96, mean age: 68.5 years, mean BMI: 26.3 kg/m^2^, % diabetes: 42.6%)	Ultrasound or computed tomography, 48.3% (*n* = 111)	Clostridium difficile‐associated diarrhoea over 4 years, 50% (*n* = 115)	Age, metabolic syndrome, chronic kidney disease, prior antibiotic use in the last 3 months and elevated serum C reactive protein levels	MASLD was associated with higher risk of Clostridium difficile‐associated diarrhoea (aOR ratio 1.51, 95% CI 1.20–1.85)	6
Papic et al., 2020^19^	Cross‐sectional hospital‐based study (retrospective analysis)	314 Croatian hospitalized adult individuals for various acute infections or sepsis (M/F: 170/144, mean age: 75 years, mean BMI: NA, % obesity: 11%, % diabetes: 28%)	Ultrasound, 26.4% (*n* = 83)	In‐hospital Clostridium difficile‐associated diarrhoea over 3 years, 9.9% (*n* = 31)	Charlson age‐comorbidity index, hospital admission within 3 months, low serum albumin, chronic kidney disease (eGFR <40 mL/min), obesity, diabetes, and use of certain antibiotics (piperacillin/tazobactam or carbapenems)	MASLD was associated with higher risk of Clostridium difficile‐associated diarrhoea (aOR ratio 3.27 95% CI 1.04–10.3)	7
Patel et al., 2024^20^	Cross‐sectional population‐based study using data from the National Inpatient Sample (NIS), the largest all‐payer USA in‐patient care database (retrospective analysis)	~26.4 million United States adult patients admitted to hospitals in 2020 (*n* = 26 432 832) (M/F: 11513311/14919521, age >65 years: 46%, >65% Caucasian, % obesity: 18.7%, % diabetes: 28.5%)	ICD‐10 codes, 3.0% (*n* = 755 910)	Acute gastrointestinal infections (as ascertained by ICD‐10 codes), .92% (*n* = 243 255). Specific rates of *Clostridium difficile*, *Escherichia coli*, and *Salmonella* were also examined	Age, sex, race, household income, income, Charlson comorbidity index, obesity, diabetes, hypertension, smoking history, dyslipidaemia, obstructive sleep apnoea, gastro‐oesophageal reflux disease and inflammatory bowel disease	MASLD was associated with higher risk of acute bacterial gastrointestinal infections (aOR 1.36, 95% CI 1.30–1.42) (principally higher infection rates from *Clostridium difficile*, *Escherichia coli*, and *Salmonella*)	6
*Prospective studies*							
Ebrahimi et al., 2023^21^	Nationwide population‐based cohort study (retrospective analysis)	Swedish adults with histologically confirmed MASLD (n = 12 133) from 1969 to 2017. Patients were matched to ≤5 population comparators (*n* = 57 516) by age, sex, calendar year and county (M/F 37771/31878, mean age 54 years, % obesity: 1.1%, % diabetes: 3%). Median follow‐up of 14.1 years	Liver biopsy, 17.5% (*n* = 12 133). NAFLD was defined as simple steatosis (*n* = 8232), non‐fibrotic steatohepatitis (*n* = 1378), non‐cirrhotic fibrosis (*n* = 1845), and cirrhosis (*n* = 678)	*n* = 19 572 cases hospitalized for serious bacterial infections over the follow‐up (especially acute respiratory, urinary tract infections and sepsis) (as ascertained by ICD‐9/ICD‐10 codes)^b^	Age, sex, county, calendar period, education level, country of birth, baseline clinical comorbidities (diabetes, obesity, dyslipidaemia, hypertension), chronic obstructive pulmonary disease and number of hospitalizations in the year preceding the index date	MASLD was associated with higher risk of developing serious bacterial infections (aHR, 1.71, 95% CI 1.63–1.79). Risk of serious infections increased with worsening histological severity of NAFLD (simple steatosis [aHR 1.64, 95% CI 1.55–1.73], non‐fibrotic steatohepatitis [aHR 1.84, 95% CI 1.60–2.12], non‐cirrhotic fibrosis [aHR 1.77, 95% CI 1.56–2.00], and cirrhosis [aHR 2.32, 95% CI,1.92–2.82]), respectively	8
Shang et al., 2023^22^	Nationwide population‐based cohort study (retrospective analysis)	Swedish adult patients with MASLD (*n* = 14 869) from 1987 to 2020 in the Swedish National Patient Register. Patients were matched to ≤10 population comparators (*n* = 137 145) by age, sex, municipality and calendar year (M/F 77276/74738, mean age 55 years, % obesity 3.5%, diabetes: 5.7%). Median follow‐up of 5.3 years	ICD codes, 9.8% (*n* = 14 869)	n = 11 889 cases hospitalized for serious infections over the follow‐up (especially acute respiratory and urogenital infections, sepsis followed by gastrointestinal infections, peritonitis and musculoskeletal or skin infections) (as ascertained by ICD‐9/ICD‐10 codes)^b^	Age, sex, municipality, calendar year, country of birth, education level, chronic obstructive pulmonary disease, cancer, rheumatic disease, inflammatory bowel disease, dementia, cardiovascular disease, diabetes, obesity and chronic kidney disease	MASLD was associated with higher risk of developing serious infections (aHR, 1.90, 95% CI 1.8–2.0). Moreover, MASLD was also associated with higher risk of death related to infections (aHR, 1.80, 95% CI 1.6–2.2). Notably, both non‐cirrhotic NAFLD (aHR 1.9, 95% CI 1.7–2.0) and NAFLD without diabetes (aHR 1.9, 95% CI 1.8–2.1) were associated with higher risk of serious infections, although this risk was stronger for NAFLD‐ cirrhosis (aHR 3.6, 95% CI 2.7–4.6). Concordant with the overall results on serious infection, all subtypes of serious infection showed a higher risk in patients with MASLD	7

Abbreviations: 25(OH)D, 25 hydroxyvitamin D; aHR, adjusted hazard ratio; ALT, alanine aminotransferase; aOR, adjusted odds ratio; BMI, body mass index; HOMA‐IR, homeostasis model assessment‐insulin resistance; MDA, malondialdehyde; NA, not available; PON‐1; paraoxonase‐1.

^a^ Originally labelled as NAFLD.

^b^ The specific ICD‐9/ICD‐10 codes used for diagnosing all subtypes of serious bacterial infections in each prospective cohort study were reported in Supplementary Documents 1 and 2 in our online‐only Supplementary Material.

### 3.2 Cross‐sectional studies on the association between MASLD and serious bacterial infections

**FIGURE 1:**
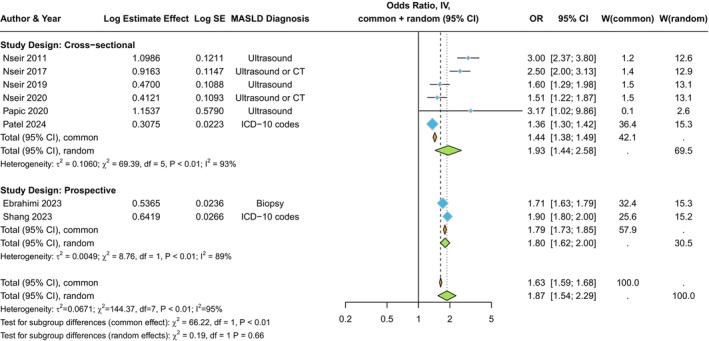
Forest plot and pooled estimates of the effect of MASLD on the risk of serious bacterial infections requiring hospital admission in the eligible studies stratified by study design (cross‐sectional vs. prospective studies).

The distribution of cross‐sectional studies (involving 26 434 377 adult individuals from different countries) by estimate of the association between MASLD and the risk of serious bacterial infections requiring hospital admission is plotted in Figure 1.^15^, ^16^, ^17^, ^18^, ^19^, ^20^ We found that MASLD was significantly associated with higher odds of serious bacterial infections (*n* = 6 studies; pooled random‐effects odds ratio 1.93, 95% CI 1.44–2.58; *I* ^*2*^ = 93%). The serious bacterial infections requiring hospital admission considered in each eligible study are reported in Table 1. Since we have always used the fully adjusted OR estimates for each eligible study, this pooled random‐effects OR was independent of age, sex, ethnicity, obesity, diabetes and other potential confounders (as specified in Table 1).

### 3.3 Prospective studies on the association between MASLD and the risk of developing serious bacterial infections

The distribution of prospective studies (*n* = 2 nationwide cohort studies including 221 663 Swedish individuals) by estimate of the association between MASLD and the risk of incident serious bacterial infections requiring hospital admission is plotted in Figure 1. MASLD assessed by liver biopsy or ICD codes was significantly associated with a higher risk of developing serious bacterial infections requiring hospital admission (pooled random‐effects hazard ratio 1.80, 95% CI 1.62–2.0; *I* ^*2*^ = 89%). This risk was independent of age, sex, county, education level and multiple baseline clinical comorbidities (including diabetes, obesity, dyslipidaemia, hypertension or chronic obstructive pulmonary disease).

### 3.4 Prospective studies on the association between the severity of MASLD and the risk of developing serious bacterial infections

**FIGURE 2:**
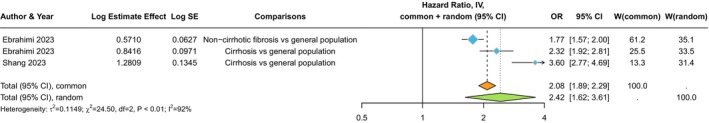
Forest plot and pooled estimates of the effect of more serious MASLD (i.e. advanced fibrosis or cirrhosis) on the risk of developing incident serious bacterial infections requiring hospital admission in the eligible prospective studies.

The distribution of prospective cohort studies by estimate of the association between the severity of MASLD and the risk of developing serious bacterial infections is plotted in Figure 2. Incidence rates of serious bacterial infections requiring hospital admission were further increased with more advanced liver disease, especially with higher fibrosis stage, that is, non‐cirrhotic fibrosis or cirrhosis (*n* = 2 studies; pooled random‐effects hazard ratio 2.42, 95% CI 1.89–2.29; *I* ^*2*^ = 92%). The ICD‐9/ICD‐10 codes used for diagnosing all subtypes of serious bacterial infections in each cohort study are specified in Supplementary Documents 1 and 2 in the online‐only Supplementary Material.

### 3.5 Subgroup analyses and meta‐regressions in cross‐sectional studies

We undertook subgroup analyses to explore the possible sources of (expected) high heterogeneity across the cross‐sectional studies. As shown in Figure S2, the association between MASLD and the odds of serious bacterial infections requiring hospital admission was consistent when the comparison was stratified by study design (case–control vs. cross‐sectional studies). Conversely, the association between MASLD and serious bacterial infections was more robust in Israeli and Croatian studies than in the USA study (Figure S3), as well as in studies where the diagnosis of MASLD was based on imaging techniques than in those using ICD codes (Figure S4).

Notably, a sensitivity analysis using the one‐study remove (leave‐one‐out) approach to test the influence of each study on the overall effect size showed that eliminating each of the cross‐sectional studies from the pooled primary analysis did not show any significant effect on the association between MASLD and risk of serious bacterial infections (Figure S5). Univariable meta‐regression analyses did not reveal any effect modification by age (Figure S6), sex (Figure S7), body mass index (Figure S8) and pre‐existing diabetes (Figure S9) on the association between MASLD and the prevalent risk of serious infections.

### 3.6 Publication bias

**FIGURE 3:**
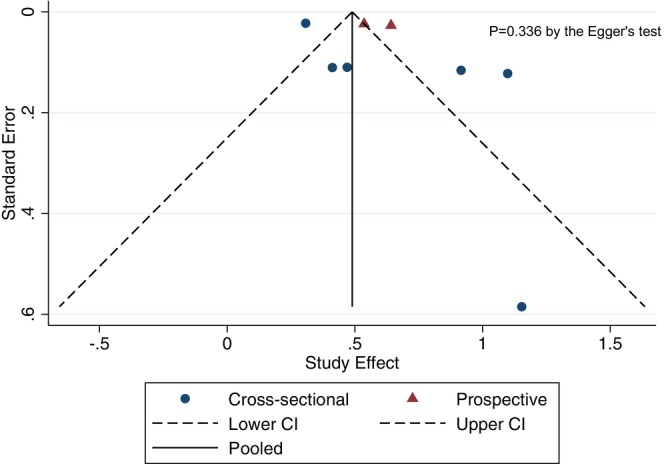
Funnel plot assessing the possibility of publication bias across the eligible studies (*n* = 8).

As shown in Figure 3, the visual inspection and the Egger's regression test (although less than 10 studies were included) did not show any statistically significant asymmetry of the funnel plot for the included studies (*p* = .336), thus suggesting that the publication bias was low.

## 4 DISCUSSION

In this comprehensive meta‐analysis that incorporated eight observational studies (six cross‐sectional hospital‐based studies and two population‐based cohort studies) with aggregate data on ~26.6 million adult individuals from different countries (Israel, Croatia, Sweden and the USA), we found that MASLD was significantly associated with a ~ twofold higher prevalence of serious bacterial infections that required in‐hospital or emergency department care (*n* = 6 studies; pooled random‐effects odds ratio 1.93, 95% CI 1.44–2.58). The association between MASLD and the risk of having serious bacterial infections requiring hospital admission remained significant in those studies where statistical analysis was adjusted for age, sex, ethnicity, obesity, type 2 diabetes and other clinical comorbidities (as specified in Table 1). Meta‐regression analyses did not show effect modification by age, sex, obesity or type 2 diabetes on the association between MASLD and the risk of serious bacterial infections. Furthermore, this association was more robust in studies where the diagnosis of MASLD was based on imaging techniques than in those using ICD codes. Meta‐analysis of data from the two population‐based cohort studies showed that MASLD assessed by liver biopsy or ICD codes was significantly associated with a ~ twofold higher incidence of serious bacterial infections requiring hospital admission (pooled random‐effects hazard ratio 1.80, 95% CI 1.62–2.0), independent of age, sex, obesity, diabetes and other baseline clinical comorbidities. Notably, the incidence rates of bacterial infections were further increased with more advanced liver disease, especially with higher fibrosis stage, that is, non‐cirrhotic fibrosis and cirrhosis (pooled random‐effects hazard ratio 2.42, 95% CI 1.89–2.29).

This is the first and most comprehensive meta‐analysis of observational studies examining the magnitude of the association between MASLD and the risk of serious bacterial infections requiring hospital admission. Concordant with the overall results on serious bacterial infections, all subtypes of bacterial infection showed a higher risk in patients with MASLD than in those without MASLD. As reported in Table 1, MASLD was associated with a substantially higher prevalence and incidence of serious bacterial infections, especially urinary tract infections, upper or lower respiratory tract infections, and gastrointestinal/abdominal infections, followed by sepsis, meningitis, peritonitis, and musculoskeletal, skin or connective tissue infections. As reported in the two Swedish cohort studies included in the meta‐analysis, there was a graded positive relationship between the severity of MASLD and the risk of developing serious bacterial infections (with the highest risk observed for patients with advanced fibrosis or cirrhosis).^21^, ^22^ Specifically, in the Swedish nationwide cohort study by Ebrahimi et al.,^21^ the risk of serious bacterial infections increased progressively and independently with worsening MASLD histological severity over a median of 14.1 years (isolated steatosis (adjusted HR 1.64, 95% CI 1.55–1.73), non‐fibrotic steatohepatitis [adjusted HR 1.84, 95% CI 1.60–2.12], non‐cirrhotic fibrosis [aHR 1.77, 95% CI 1.56–2.0] and cirrhosis [adjusted HR 2.32, 95% CI 1.92–2.82]). Similarly, in the cohort study by Shang et al.,^22^ both non‐cirrhotic MASLD (adjusted HR 1.90, 95% CI 1.7–2.0) and MASLD without diabetes (adjusted HR 1.90, 95% CI 1.8–2.1) were independently associated with higher risk of developing serious bacterial infections, although this risk was stronger for MASLD‐related cirrhosis (adjusted HR 3.60, 95% CI 2.7–4.6). In this latter study,^22^ MASLD was also associated with a higher risk of infection‐related mortality (adjusted HR 1.80, 95% CI 1.6–2.2). Based on subgroup analyses, these investigators also noted that attention should be paid to young and female patients with MASLD, whose liver disease may have a comparatively stronger adverse impact on the incidence of severe bacterial infections.^22^ The findings of our meta‐analysis complement also the results of previously published meta‐analyses showing that MASLD was associated with an increased risk of *H. pylori* infection and more severe COVID‐19 infection.^23^, ^24^

The findings of our meta‐analysis may have some important clinical implications. These results apply to serious bacterial infections requiring in‐hospital or emergency department care and, therefore, cannot be extrapolated to milder bacterial infections. That said, clinicians need to be aware of the increased risk in most, if not all, types of bacterial infections among patients with MASLD (especially among those with advanced fibrosis or cirrhosis) and should also have increased clinical vigilance for serious bacterial infections and consider preventive measures, such as regular checks of vaccination status.^21^, ^22^ Patients should be advised to seek medical attention in case of acute infections. With an approximately twofold increase in the risk of developing serious bacterial infections and infection‐related death, patients with MASLD should receive broad‐spectrum antibiotics if hospitalized for bacterial infection, with the choice of antibiotic therapy refined when culture test results become available.^25^ Furthermore, given the greater risk of colonization by multidrug‐resistant bacteria (MDR bacteria) in patients with liver failure, it could also be helpful to perform rectal and/or nasal swabs for MDR bacteria in the presence of serious infection. Future studies should examine whether drug treatment of MASLD will reduce the incidence rates of serious bacterial infections in this patient population. Although not specifically tested in the present meta‐analysis, it is also reasonable to assume that patients with MASLD, especially those with advanced MASLD or with coexisting diabetes/obesity, are at increased risk of developing viral, fungal and parasitic infections. This implies that clinicians need to be aware that the risk of infectious diseases is high in this patient population and should be monitored especially in patients with MASLD‐related cirrhosis and those who have coexisting type 2 diabetes or obesity.

A detailed discussion of the putative mechanisms underpinning the association between MASLD and the risk of serious bacterial infections (e.g. pneumonia, meningitis, urinary tract infections, gastrointestinal/abdominal infections, peritonitis and sepsis) is beyond the scope of the meta‐analysis. Briefly, the liver is essential for host defence against infectious diseases. Bacterial infections are common among patients with cirrhosis (including spontaneous bacterial peritonitis) and may result in hepatic decompensation and liver‐related mortality.^26^ MASLD is closely associated with obesity and type 2 diabetes. While these are two metabolic diseases known to increase the susceptibility to bacterial infections (and other types of infections), neither obesity nor pre‐existing type 2 diabetes modified the strength of the association in univariable meta‐regression analyses we undertook. Other MASLD‐related factors, such as systemic low‐grade chronic inflammation and impaired immune system function, may also contribute to the increased susceptibility to bacterial infections in patients with MASLD.^25^, ^27^ Accumulating research also supports a role of intestinal barrier disruption and bacterial translocation from leaky gut in MASLD as an important contributing factor to development of bacterial infections and other infection types (viral, fungal or parasitic), both at the intestinal and extra‐intestinal level.^28^ However, the precise mechanisms and the interplay of the mentioned factors remain not fully understood. Further mechanistic studies are needed to better elucidate these issues.

Our meta‐analysis has some important limitations, which are inherent to the design of the included studies. First, since most of the included studies had a retrospective cross‐sectional design, we cannot establish a causal association between MASLD and serious bacterial infections requiring hospital admission. Second, although most eligible studies adjusted the results for age, sex, ethnicity, obesity, diabetes and other clinical comorbidities, the possibility of residual confounding by unmeasured factors cannot be entirely excluded. For instance, although the diagnosis of MASLD requires the exclusion of significant alcohol consumption, alcohol consumption is often under‐reported in individuals classified as having MASLD (thus inducing a possible misclassification bias) and alcohol and metabolic factors may interact to exacerbate the progression of liver disease and the risk of developing bacterial infections.^29^ Third, although we used a random‐effects model, the interpretation of some results requires some caution because of the observed medium‐high heterogeneity for the pooled primary analysis of cross‐sectional studies and the moderate overall quality of the studies, suggesting a medium‐high risk of bias according to the NOS. Detailed subgroup analyses by types of serious bacterial infection (that could partly explain the observed high between‐study heterogeneity) cannot be performed. Fourth, although all eligible studies have used the NAFLD nomenclature, for this meta‐analysis, we have assumed that NAFLD and MASLD are synonymous, as the two fatty liver disease nomenclatures share a very high level of overlap (>96% of individuals with NAFLD meet the diagnostic criteria for MASLD).^30^, ^31^, ^32^ Fifth, an assessment of the severity of liver fibrosis (using non‐invasive fibrosis biomarkers/scores) was not available for the eligible cross‐sectional studies; it was only available for the two prospective cohort studies. Sixth, although a selective reporting bias of eligible studies could not be excluded, our comprehensive search has made it unlikely that any published studies were missed. Sixth, although in one large cross‐sectional study by Patel et al. the diagnosis of NAFLD was based on ICD codes (so the liver disease could be underdiagnosed), it should be noted that the one‐study remove (leave‐one‐out) approach to test the influence of each study on the overall effect size showed that eliminating of the study by Patel et al. (Figure S5) from the pooled analysis increased the magnitude of the association between MASLD and risk of serious bacterial infections (pooled random‐effects odds ratio 2.11, 95% CI 1.55–2.88). Finally, these results derived from studies from high‐income countries may not be directly transferable to low‐income countries (where the risk of severe bacterial infections is higher) because poor communities were largely underrepresented in the meta‐analysis. Further prospective cohort studies will be needed in low‐ and lower‐middle‐income countries.^33^

Despite these limitations, the present meta‐analysis also has important strengths. Our meta‐analysis is the first and largest assessment of the association between MASLD and the risk of serious bacterial infections requiring hospital admission. The large number of individuals included in the meta‐analysis (although the limited number of observational studies available so far) provided sufficient statistical power to quantify the magnitude of the association between MASLD and the risk of serious bacterial infections. Moreover, we have used standardized risk estimates from all included studies to allow a consistent combination of estimates across eligible studies. Finally, the visual inspection of the funnel plot did not reveal any significant asymmetry, thus suggesting that the risk of publication bias was low.

In conclusion, this comprehensive meta‐analysis provides evidence of a significant association between MASLD and an increased risk of serious bacterial infections requiring hospital admission (such as pneumonia, meningitis, urinary tract infections, gastrointestinal/abdominal infections or sepsis). This association remained statistically significant in those studies whose results were adjusted for age, sex, ethnicity, obesity, type 2 diabetes and other important clinical comorbidities. Incidence rates of serious bacterial infections increased further with greater severity of MASLD, principally higher fibrosis stage. Future well‐designed large prospective cohort studies from different countries (including Asian populations and low‐income countries) are needed to further corroborate these results, and mechanistic studies are also required to better decipher the complex molecular pathways linking MASLD with increased susceptibility to developing serious bacterial infections requiring hospital admission.

## AUTHOR CONTRIBUTIONS

All listed authors contributed greatly to this study and fulfilled the stated guidelines for authorship, as described by the published criteria for authorship by the ICMJE. Each author participated sufficiently in the work to take public responsibility for appropriate portions of the content. Below is a detailed account of each author's contribution: AM and GT were involved in the conception of the study and the analysis and interpretation of the results. GT wrote the first draft of the manuscript. RM, VF and MGL were involved in the conduct of the study and searched the published articles. AG, LM, FG, HT and CDB were involved in the interpretation of the results and contributed to the discussion. All authors edited, reviewed and approved the final version of the manuscript.

## FUNDING INFORMATION

There was no funding source for this specific study. GT is supported in part by grants from the University School of Medicine of Verona, Verona, Italy. CDB is supported in part by the Southampton NIHR Biomedical Research Centre (NIHR203319), UK. This study was partly supported by the Italian Ministry of Health under Fondi Ricerca Corrente — Linea 1 (IRCCS Sacro Cuore ‐ Don Calabria Hospital).

## CONFLICT OF INTEREST STATEMENT

The authors have no potential conflicts of interest to disclose.

## ETHICS STATMENT

This study involves human participants but was not approved by an Ethics Committee. Approval from an Ethics Committee is not necessary as this is a meta‐analysis of published observational studies that had already obtained both informed consent from participants and ethical approval by their local Ethics committees.

## Supporting information


Data S1:

## Data Availability

All supporting data of the meta‐analysis are available within the article and in the online‐only Supplementary Material.
