# Supplementary material for: Association between MASLD and increased risk of serious bacterial infections requiring hospital admission: A meta‐analysis
Source: Liver Int. 2024 Sep 11;45(4):e16101. doi: 10.1111/liv.16101 (PMC11892334; doi:10.1111/liv.16101)
Supplement: Supplementary file 1 — Data S1: [file LIV-45-0-s001.pdf]

Mantovani A, Morandin R, Fiorio V, et al. Association Between Metabolic Dysfunction-Associated Steatotic Liver Disease and Increased Risk of Serious Bacterial Infections Requiring Hospital Admission: A Meta-Analysis.

ONLINE-ONLY SUPPLEMENTARY MATERIAL

Supplementary Table 1. Observational studies *excluded* at the stage of eligibility according to the PRISMA flow diagram.

Supplementary Figure 1. The PRISMA flow diagram for search and selection processes of the meta-analysis.

Supplementary Figure 2. Forest plot and pooled estimates of the effect of MASLD on the risk of serious bacterial infections requiring hospital admission in the eligible cross-sectional studies stratified by study design (cross-sectional vs. case-control).

Supplementary Figure 3. Forest plot and pooled estimates of the effect of MASLD on the risk of serious bacterial infections requiring hospital admission in the eligible cross-sectional studies stratified by study country.

Supplementary Figure 4. Forest plot and pooled estimates of the effect of MASLD on the risk of serious bacterial infections requiring hospital admission in the eligible cross-sectional studies stratified by methodologies used for diagnosing MASLD.

Supplementary Figure 5. One-study remove ("leave-one-out") analysis to test the influence of each study on the overall effect size of the effect of MASLD on the risk of serious bacterial infections requiring hospital admission in the eligible cross-sectional studies.

Supplementary Figure 6. Bubble plot with a fitted meta-regression line (in blue) showing the pooled estimates of the effect of age on the odds of serious bacterial infections requiring hospital admission in cross-sectional studies.

Supplementary Figure 7. Bubble plot with a fitted meta-regression line (in blue) showing the pooled estimates of the effect of male sex on the odds of serious bacterial infections requiring hospital admission in cross-sectional studies.

Supplementary Figure 8. Bubble plot with a fitted meta-regression line (in blue) showing the pooled estimates of the effect of body mass index on the odds of serious bacterial infections requiring hospital admission in cross-sectional studies.

Supplementary Figure 9. Bubble plot with a fitted meta-regression line (in blue) showing the pooled estimates of the effect of pre-existing type 2 diabetes on the odds of serious bacterial infections requiring hospital admission in cross-sectional studies.

Supplementary Document 1. Specific ICD codes used for diagnosing all subtypes of serious bacterial infections requiring hospital admission in the cohort study by Ebrahimi F et al. (Ebrahimi F, Simon TG, Hagström H, et al. Risk of Severe Infection in Patients With Biopsy-proven Nonalcoholic Fatty Liver Disease – A Population-based Cohort Study. Clin Gastroenterol Hepatol. 2023;21(13):3346-3355.e19. doi:10.1016/j.cgh.2023.05.013).

Supplementary Document 2. Specific ICD codes used for diagnosing all subtypes of serious bacterial infections requiring hospital admission in the cohort study by Shang Y et al. (Shang Y, Widman L, Ebrahimi F, Ludvigsson JF, Hagström H, Wester A. Risk of infections in non-alcoholic fatty liver disease: A nationwide population-based cohort study. Liver Int. 2023;43(10):2142-2152. doi:10.1111/liv.15680).

Supplementary Table 1. Observational studies *excluded* at the stage of eligibility according to the PRISMA flow diagram.

Author, year	Study design	Main reason(s) for exclusion
Nseir W et al., 2019 (PMID: 30325458)	Cross-sectional hospital-based study (Israel)	Unsatisfactory study outcome (the study examined the association between MASLD and the risk of 30-day all-cause death in patients with community-acquired pneumonia)
Jiang Y et al., 2021 (PMID: 34904045)	Cross-sectional hospital-based study (from nationwide inpatient sample database in USA)	Unsatisfactory study outcome (the study examined the impact of MASLD on the risk of in-hospital mortality and complications in patients with Clostridium difficile-associated diarrhea)
Gjurasin B et al., 2023 (PMID: 36675985)	Cross-sectional hospital-based study (Croatia)	Unsatisfactory study outcome (the study examined the impact of MASLD on the risk of in-hospital death and complications in patients with severe community-acquired pneumonia)
Krznaric J et al., 2024 (PMID: 38337491)	Cross-sectional hospital-based study (Croatia)	Unsatisfactory study outcome (the study examined the impact of MASLD on the risk of in-hospital mortality and complications in patients with community-acquired sepsis)

References to the table

1. Nseir WB, Mograbi JM, Amara AE, Abu Elheja OH, Mahamid MN. Non-alcoholic fatty liver disease and 30-day all-cause mortality in adult patients with community-acquired pneumonia. QJM. 2019;112(2):95-99. doi:10.1093/qjmed/hcy227.
2. Jiang Y, Chowdhury S, Xu BH, et al. Nonalcoholic fatty liver disease is associated with worse intestinal complications in patients hospitalized for Clostridioides difficile infection. World J Hepatol. 2021;13(11):1777-1790. doi:10.4254/wjh.v13.i11.1777.
3. Gjurašin B, Jeličić M, Kutleša M, Papić N. The Impact of Nonalcoholic Fatty Liver Disease on Severe Community-Acquired Pneumonia Outcomes. Life (Basel). 2022;13(1):36. Published 2022 Dec 23. doi:10.3390/life13010036.
4. Krznaric J, Papic N, Vrsaljko N, Gjurasin B, Kutlesa M, Vince A. Steatotic Liver Disease and Sepsis Outcomes-A Prospective Cohort Study (SepsisFAT). J Clin Med. 2024;13(3):798. Published 2024 Jan 30. doi:10.3390/jcm13030798.

Supplementary Table 1. Observational studies *excluded* at the stage of eligibility according to the PRISMA flow diagram.

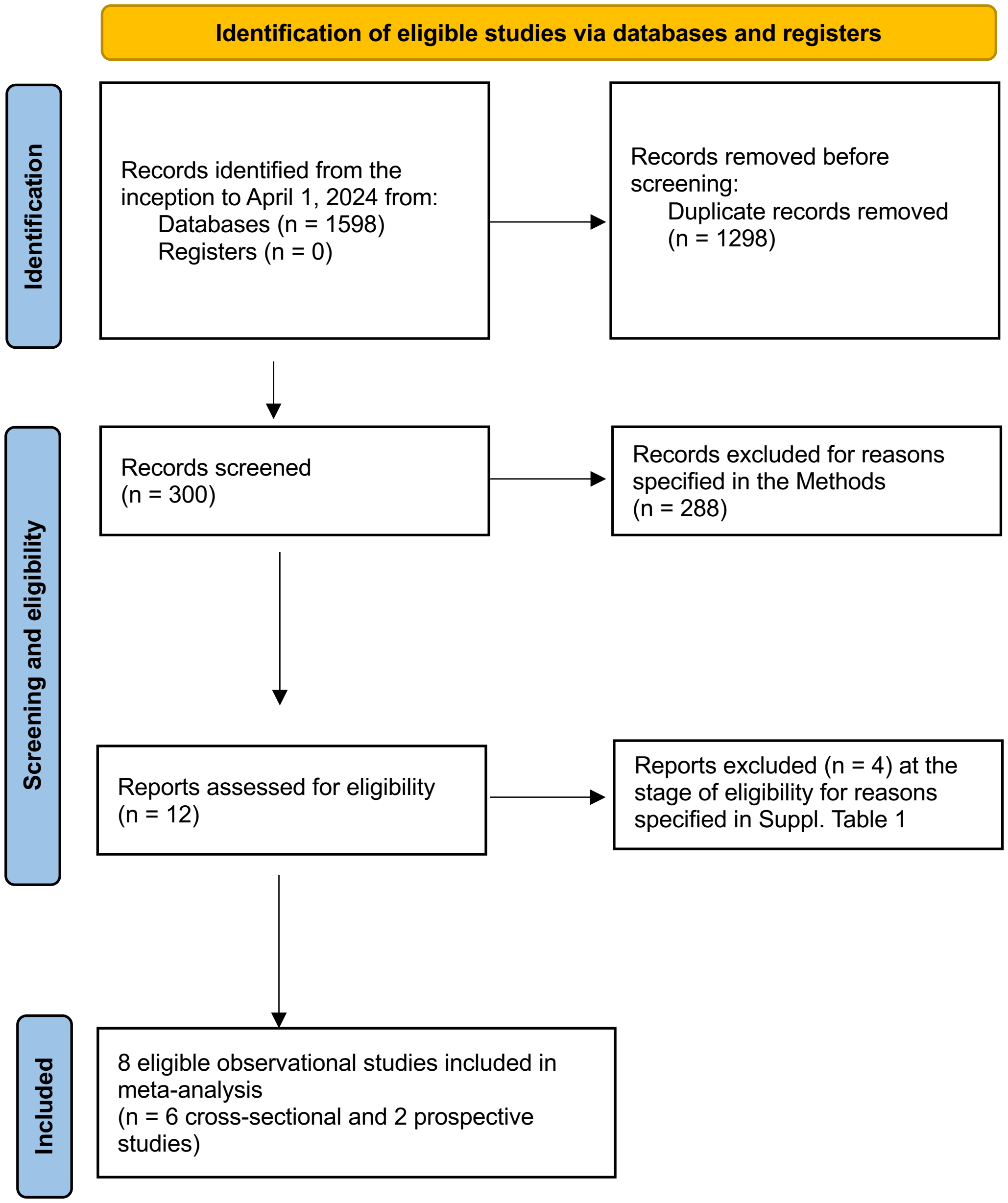

Supplementary Figure 2. Forest plot and pooled estimates of the effect of MASLD on the risk of serious bacterial infections requiring hospital admission in the eligible cross-sectional studies stratified by study design (cross-sectional vs. case-control).

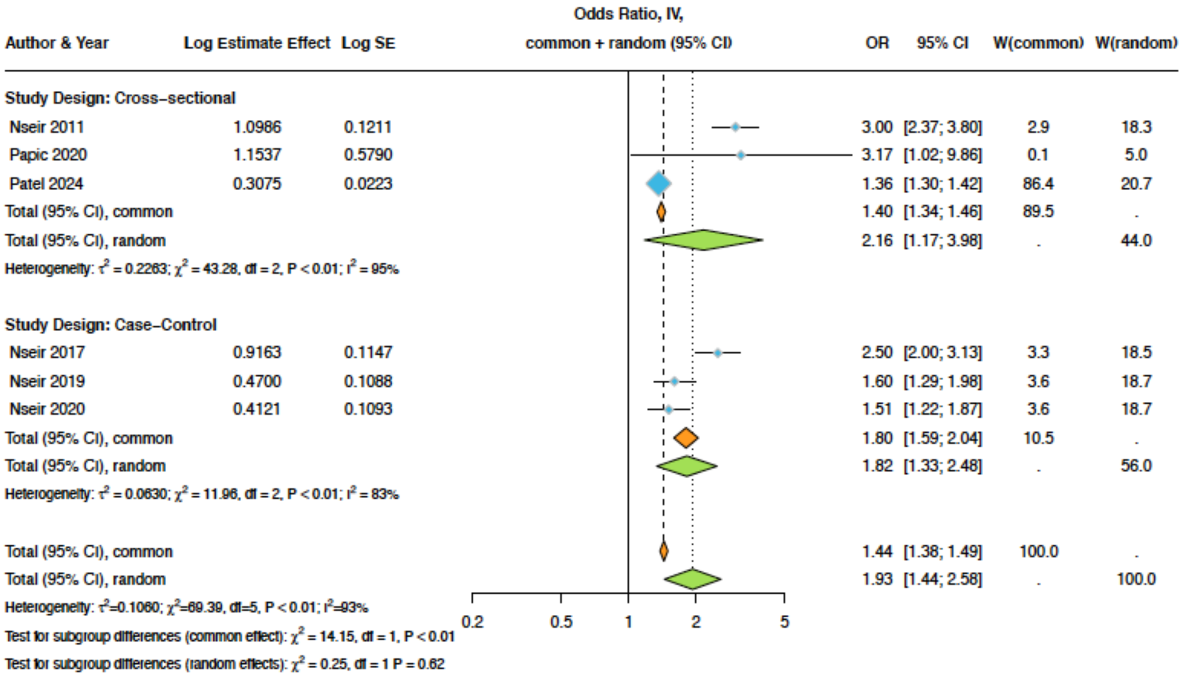

Supplementary Figure 3. Forest plot and pooled estimates of the effect of MASLD on the risk of serious bacterial infections requiring hospital admission in the eligible cross-sectional studies stratified by study country.

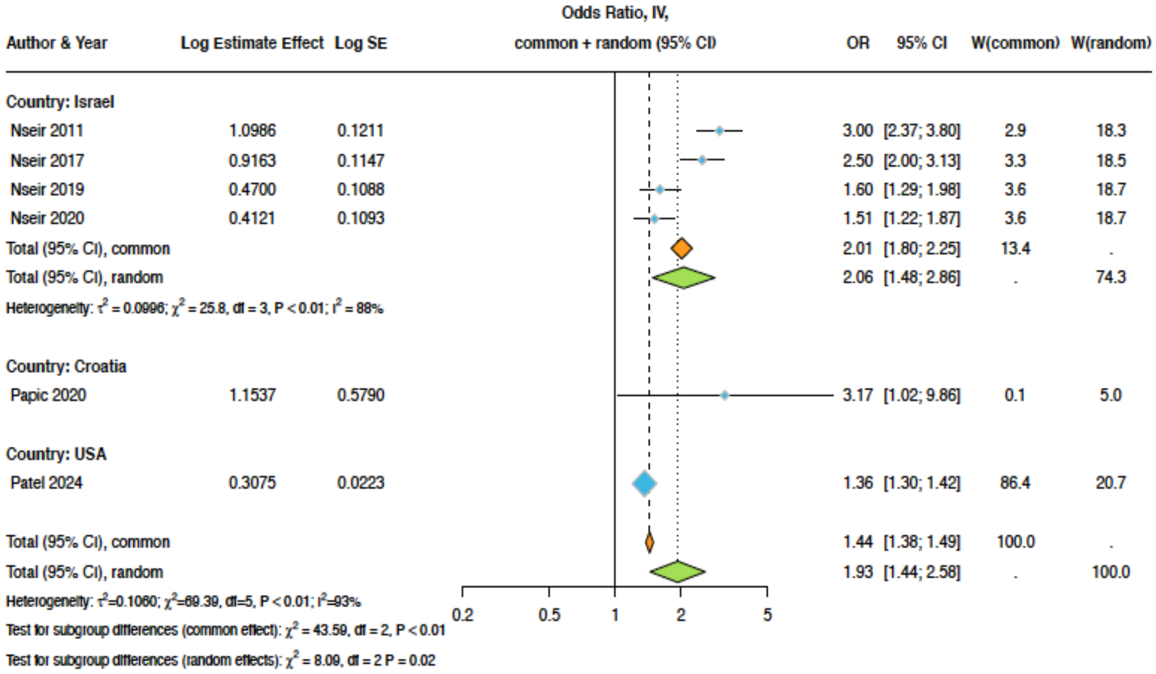

Supplementary Figure 4. Forest plot and pooled estimates of the effect of MASLD on the risk of serious bacterial infections requiring hospital admission in the eligible cross-sectional studies stratified by methodologies used for diagnosing MASLD.

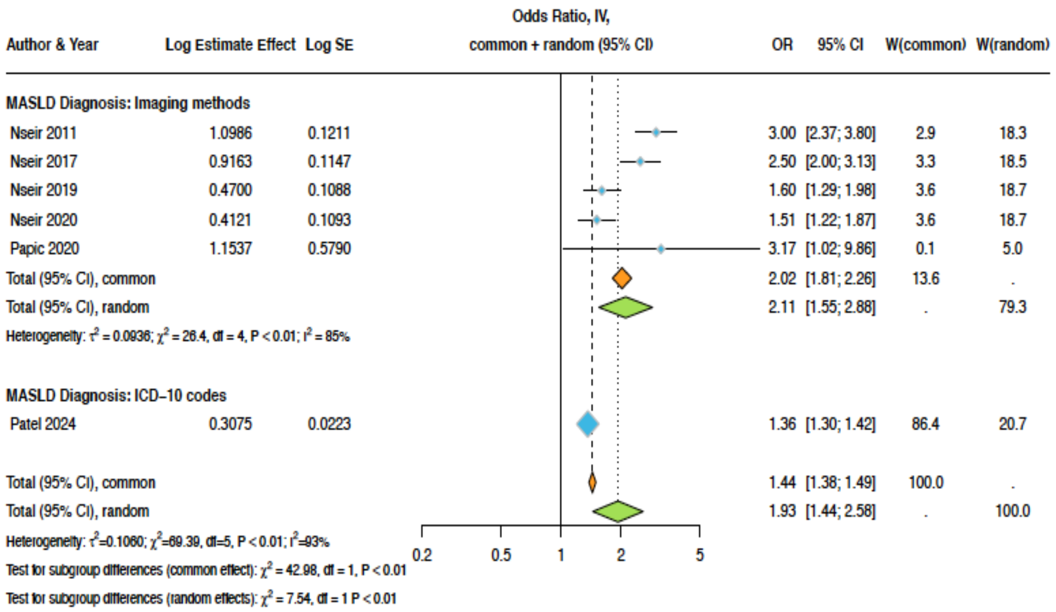

Supplementary Figure 5. One-study remove ("leave-one-out") analysis to test the influence of each study on the overall effect size of the effect of MASLD on the risk of serious bacterial infections requiring hospital admission in the eligible cross-sectional studies.

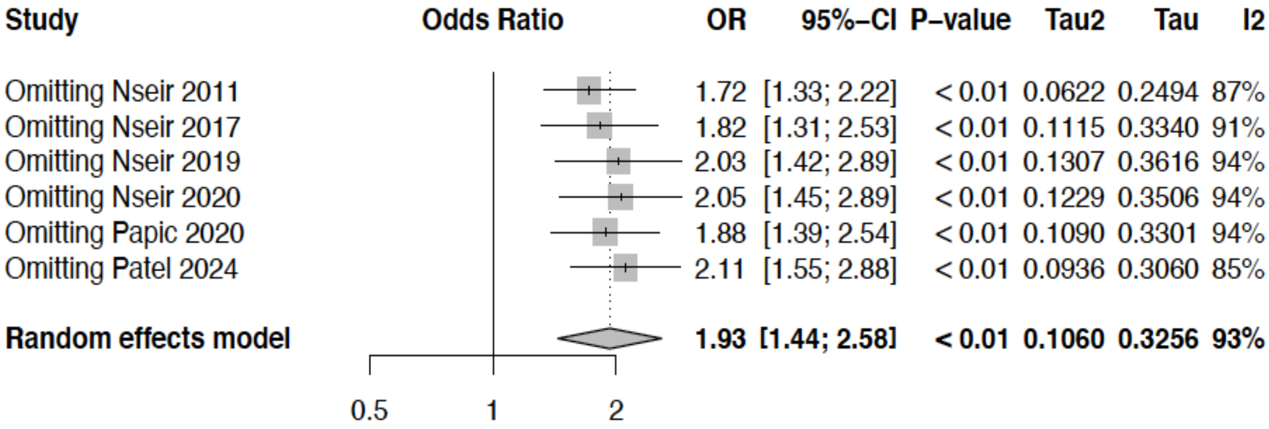

Supplementary Figure 6. Bubble plot with a fitted meta-regression line (in blue) showing the pooled estimates of the effect of age on the odds of serious bacterial infections requiring hospital admission in cross-sectional studies.

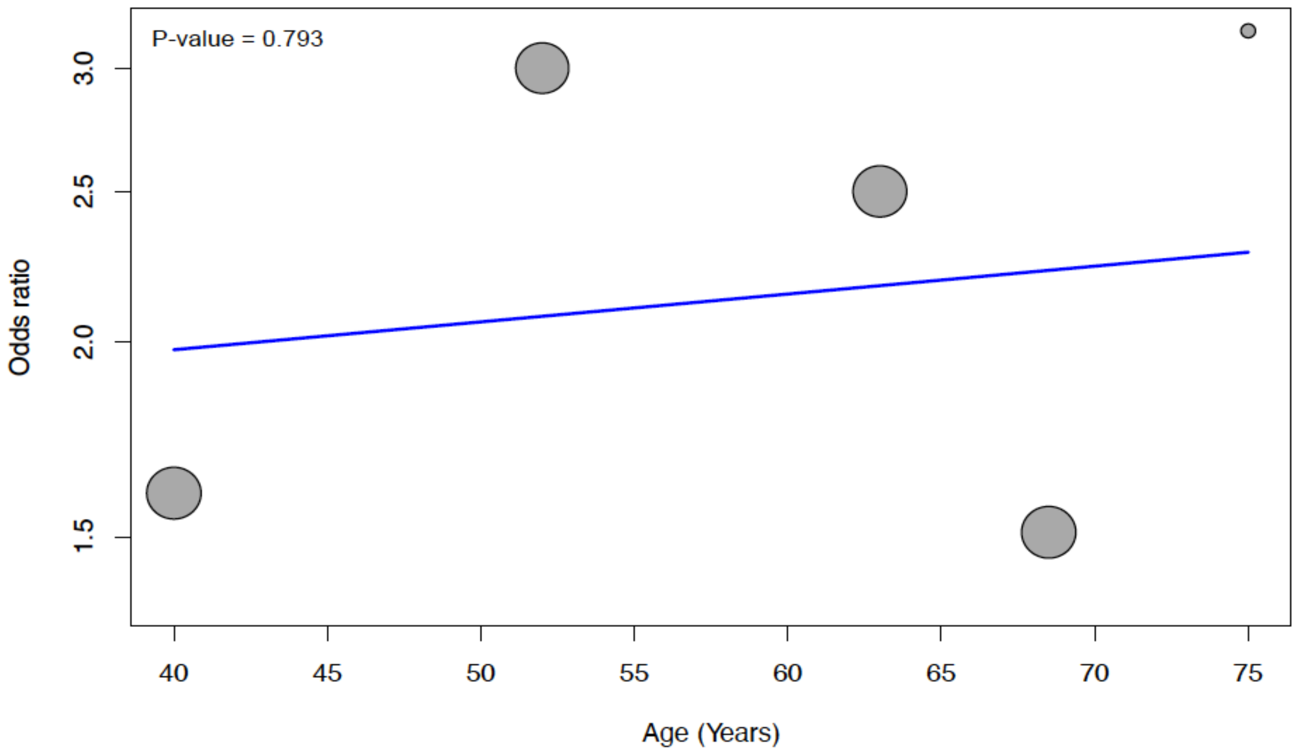

Supplementary Figure 7. Bubble plot with a fitted meta-regression line (in blue) showing the pooled estimates of the effect of male sex on the odds of serious bacterial infections requiring hospital admission in cross-sectional studies.

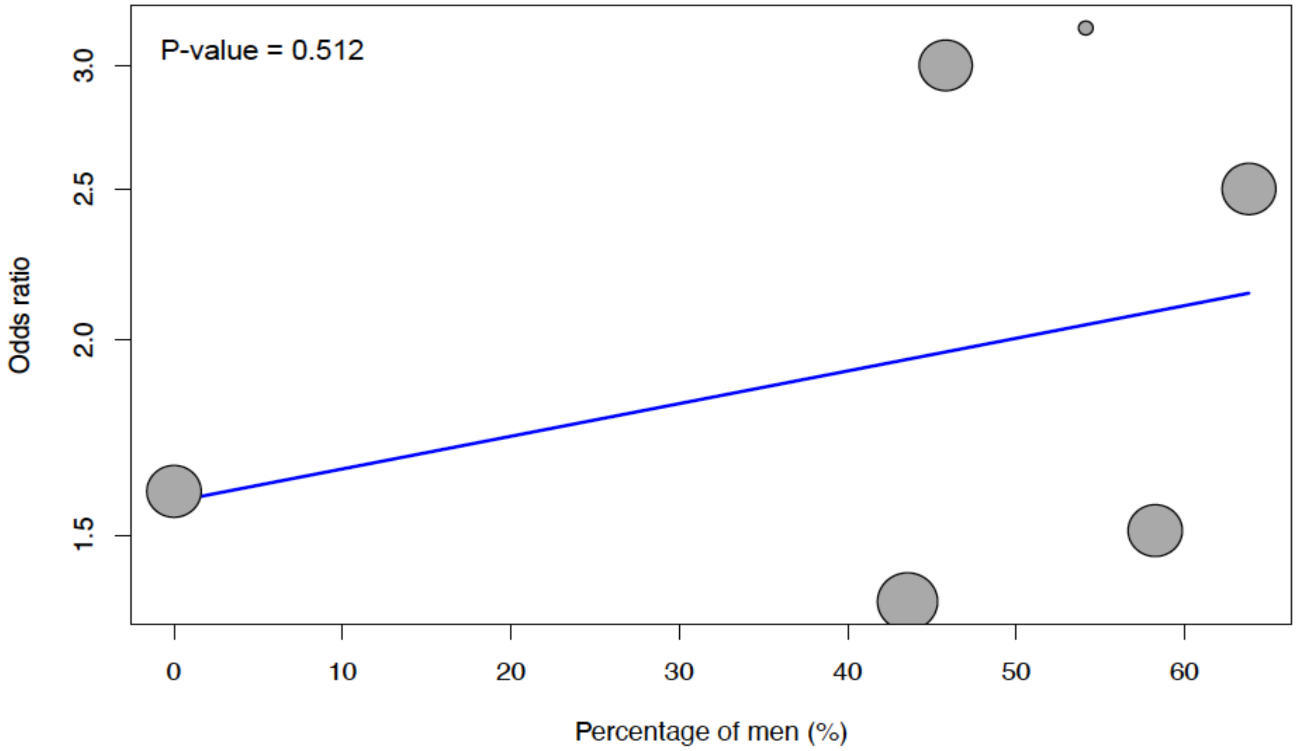

Supplementary Figure 8. Bubble plot with a fitted meta-regression line (in blue) showing the pooled estimates of the effect of body mass index on the odds of serious bacterial infections requiring hospital admission in cross-sectional studies.

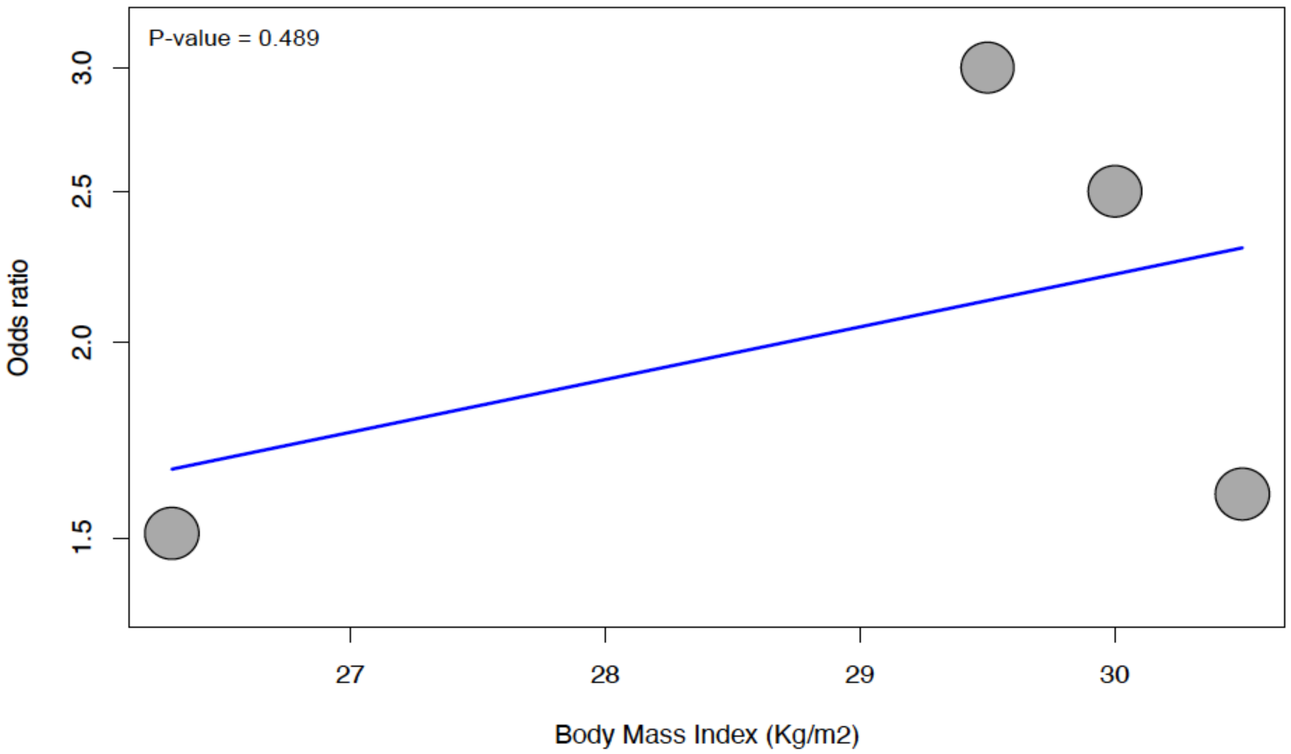

Supplementary Figure 9. Bubble plot with a fitted meta-regression line (in blue) showing the pooled estimates of the effect of pre-existing type 2 diabetes on the odds of serious bacterial infections requiring hospital admission in cross-sectional studies.

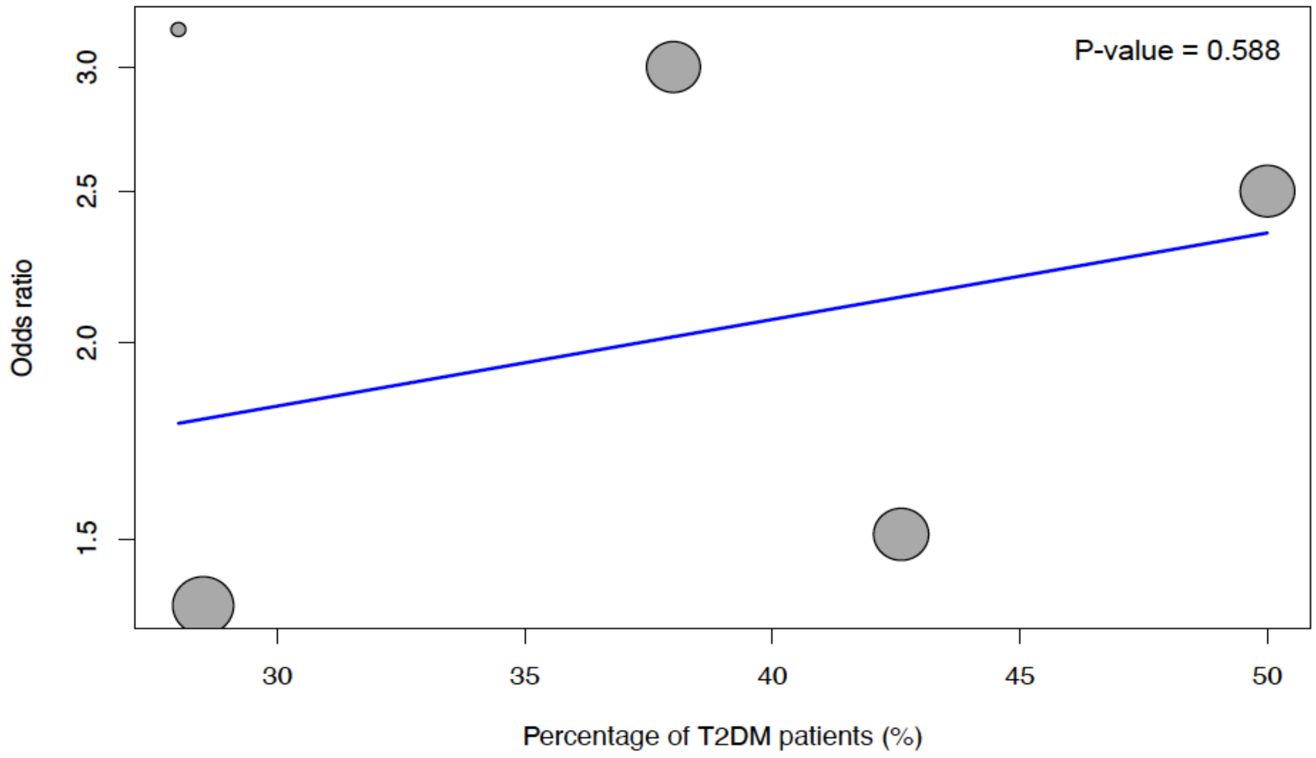

Supplementary Document 1. Specific ICD codes used for diagnosing all subtypes of serious bacterial infections requiring hospital admission in the cohort study by Ebrahimi F et al.

(Ebrahimi F, Simon TG, Hagström H, et al. Risk of Severe Infection in Patients With Biopsy-proven Nonalcoholic Fatty Liver Disease – A Population-based Cohort Study. Clin Gastroenterol Hepatol. 2023;21(13):3346-3355.e19. doi:10.1016/j.cgh.2023.05.013).

Supplementary Table 3. Definition of Primary Endpoint Any Severe Infection and Infection Subcategories

Infection	ICD-8 code	ICD-9 code	ICD-10 code
Sepsis			
Sepsis	038	038	A 39.2, A40-41, R65.1
Septic shock		785F	R57.2
Anaerobic sepsis		038D	A41.4
Gram negative sepsis	038.80	038E	A41.5
Haemophilus influencae sepsis			A41.3
Listeria sepsis			A32.7
Candida sepsis		112F	B37.7
Meningococcal sepsis	036.10,80, 97,99	036C-X	A39.2-9
Pneumococcal sepsis	038.20	038C	
Salmonella sepsis/ (para) typhoid fever	001-002	002, 003B	A02.1
Staphylococcal sepsis (including TSS)	038.10	038B	A41.0-2, A48.3
Streptococcal sepsis	038.00	038A	A40
Respiratory tract infections (including ENT)			
Bronchitis and bronchiolitis	466,99	466	J20, J21
Tuberculosis	011-019	010-018, 320E	A15-A19, K23.0, K93.0
Chronic obstructive lung disease with infection			J44.0
Inflammation and abscesses in salivary glands, mouth, tongue	527.30, 528.30, 529.00	527C,D, 528D, 529A	K11.2-3, K12.2, K14.0
Laryngitis, tracheitis and epiglottitis	464, 508.03	464	J04, J05
Mastoiditis, petrositis	382.00,99 383.00,99	383A,C,X	H70.0, H70.2, H70.9, H75.0
Nasal abscess	508.01	478B	J34.0
Other lower respiratory tract infection			J22
Otitis (including external)	380, 381.00,99, 382.00,99	380B, 382A,E,X	H60.0-3 H62.0-4, H66, H67.0-1
Parotitis	072	072	B26
Peritonsillar, pharyngeal and retropharyngeal abscess	501.99, 508.02	475, 478C	J36, J39.0-1
Pertussis	033	033	A37
Pharyngitis	074.00-01, 462	074A, 462, 034A	B08.5, J02
Pleural empyema	510	510	J86
Pleuritis	511.10-20	511A,B, X	
Pneumonia (all: viral, bacterial, fungal)	480.99 (virus) 481-484, 485.09, 486	480 (virus), 481, 482, 483, 484, 485, 486	J12 (Virus), J13, J14, J15, J16, J17, J18
Pulmonary abscess	513.99	006E, 513	A06.5, J85
Sinusitis including ethmoiditis	461	461	J01
Tonsillitis	034.00, 463	034A, 463	J03
Unspecified respiratory tract infection			J98.7
Upper respiratory tract infection	465.99	465	J06
Gastrointestinal/abdominal infections excluding SBP			
Gastroenteritis –bacterial/protozoal	001-004, 006-007 (excl 006.00), 008.00-008.30	001-004, 006A-C, W,X, 007, 008A-F	A00-04 A06-07 (excl A06.4-6)
Gastroenteritis – unspecified	009	008W, 009	A09
Intestinal abscess	569.00	569F	K63.0
Liver abscess (including amoeba) /liver infection	006.00, 572.99	006D, 572A	A06.4, K75.0(?), K77.0
Perianal/anal abscess	566	566	K61
Bacterial peritonitis including SBP			
Peritonitis including SBP	567	567A,B,C,X	K65.0,9, K67
Urogenital infections			
Cystitis/urethritis	595.00,09 597.00,09	595A,W,X, 597	N30.0, N30.8-9, N33, N34.0-1
Glomerular, tubulointerstitial , disease (from infection) including pyelonephritis	590.10-14	078G, 590B,D,W,X	N08.0, N10, N12, N13.6, N16.0, A98.5
Hydrocele (infected)		603B	N43.1
Pelvic infection	567.00 616.00, 02		N74

Supplementary Document 2. Specific ICD codes used for diagnosing all subtypes of serious bacterial infections requiring hospital admission in the cohort study by Shang Y et al.

(Shang Y, Widman L, Ebrahimi F, Ludvigsson JF, Hagström H, Wester A. Risk of infections in non-alcoholic fatty liver disease: A nationwide population-based cohort study. Liver Int. 2023;43(10):2142-2152. Doi:10.1111/liv.15680)

	**ICD10**	**ICD9**
Any infection	A00-A05, A09, A15-A58, A65-A79, B95-96, B99, D73.3, E06.0, G00-01, G06-07, H10, H44.0, H60.0-3, H62.0, H66, H67.0, H70.0, H70.2, H70.9, H75.0, I00-I01, I30.1, I32.0-1, I33.0, I40.0, I41.0, I43.0, I52.0, J01-06, J13-16, J17.0, J20.0-2, J34.0, J36, J39.0-1, J44.0, J85, J86, K11.3, K12.2, K14.0, K23.0, K61, K63.0, K65.0, K65.9, K67, K75.0, K93.0, L00-L05, L08, L30.3, M00, M01, M46.2/3/5, M49.0-3, M60.0, M63.0, M63.2, M65.0/1, M68.0, M72.6, M86.0-2, M86.9, M90.0-2, N08.0, N10, N12, N13.6 N15.1, N15.9, N16.0, N29.1, N30.0/8/9, N33, N34.0-1, N39.0, N43.1, N74, O85, O91, R57.2, R65.1	001-004, 008A-F, 008W, 009-018, 020-027, 030-038, 040-041, 073, 078J, 080-083, 087, 100-104, 136D, 245A, 254B, 320E, 360A, 373B, 376A, 380B, 382, 383A, 383C, 383X, 390-391, 421, 461, 462-466, 475, 478B, 478C, 481-483, 484C-F, 484W, 485, 486, 510, 511B, 513, 527D, 528D, 529A, 566, 567A-C, 567X, 569F, 572A, 590B-X, 595A/W/X, 597, 598A, 599A, 603B, 611A, 659D, 672, 680-686, 711A, 728A, 730A/C/X, 785F
Infection subtypes		
Sepsis	A02.1, A32.7, A39.2, A40, A41, A48.3, R57.2, R65.1	002, 003B, 036C, 038, 659D, 785F
Ear-nose-throat or respiratory tract infections	A15-19, A37, H60.0-3, H62.0, H66, H67.0, H70.0, H70.2, H70.9, H75.0, K11.3, K12.2, K14.0, J01-06, J13-16, J17.0, J20.0-2, J34.0, J36, J39.0-1, J44.0, J85, J86	010-018, 033, 034, 320E, 380B, 382, 383A, 383C, 383X, 461, 462-466, 475, 478B, 478C, 481-483, 484C-F, 484W, 485, 486, 510, 511B, 513, 527D, 528D, 529A
Gastrointestinal/abdominal infections excluding peritonitis	A00-A04, A09, K23.0, K61, K63.0, K75.0, K93.0	001-004, 008A-F, 008W, 009, 566, 569F, 572A
Peritonitis including spontaneous bacterial peritonitis	K65.0, K65.9, K67	567A-C, 567X
Urogenital infections	N08.0, N10, N12, N13.6 N15.1, N15.9, N16.0, N29.1, N30.0/8/9, N33, N34.0-1, N39.0, N43.1, N74	078J, 590B-X, 595A/W/X, 597, 598A, 599A, 603B
Musculoskeletal, skin, and connective tissue infections	A46, M00, M01, M46.2/3/5, M49.0-3, M60.0, M63.0, M63.2, M65.0/1, M68.0, M72.6, M86.0-2, M86.9, M90.0-2, L00-L05, L08, L30.3	035, 680-686, 711A, 728A, 730A/C/X
